# Shared Burden: Public Health Impact of Nine Environmental Pollutants in Europe

**DOI:** 10.1289/ehp.122-A136

**Published:** 2014-05-01

**Authors:** Lindsey Konkel

**Affiliations:** Lindsey Konkel is a Worcester, MA–based journalist who reports on science, health, and the environment. She is an editor for Environmental Health News and The Daily Climate.

Airborne particulate matter may be the most significant environmental risk factor for disease across much of Western Europe, according to a new analysis published this month in *EHP*.^1^ The study compares the disease burden associated with nine environmental exposures across six European countries—Finland, Germany, France, Italy, Belgium, and the Netherlands. 

Few studies have sought to rank environmental risk factors on an international level by their public health impact—a critical aspect for developing effective policy measures, according to the team led by Otto Hänninen, a pollution exposure researcher at Finland’s National Institute for Health and Welfare. “Comparability across countries is very useful in promoting abatement that requires international policies—such as those pertaining to long-range transported particulate matter or vehicle emissions,” Hänninen says.

The research team evaluated the potential public health impact for each of the nine exposures by estimating years of life lost due to death and disability. For each exposure, they selected one or more health outcomes that had been causally associated with it—for instance, cardiac death and chronic bronchitis associated with fine particulate matter (PM_2.5_), sleep disturbance associated with traffic noise, onset of asthma associated with secondhand smoke exposure, and cancer resulting from exposure to dioxins and dioxin-like polychlorinated biphenyls (PCBs).^2^ Other environmental risk factors assessed included benzene, formaldehyde, lead, ozone, and radon.

Hänninen and colleagues concluded that about 3–7% of the total annual disease burden across all six countries could be associated with these nine environmental exposures. PM_2.5_ was the environmental risk factor with the highest public health significance, accounting for 68% of the total estimated health impacts. Secondhand smoke and traffic noise tied for second place, each estimated to account for 8% of the environmental disease burden, while radon accounted for an estimated 7%.

**Figure:**
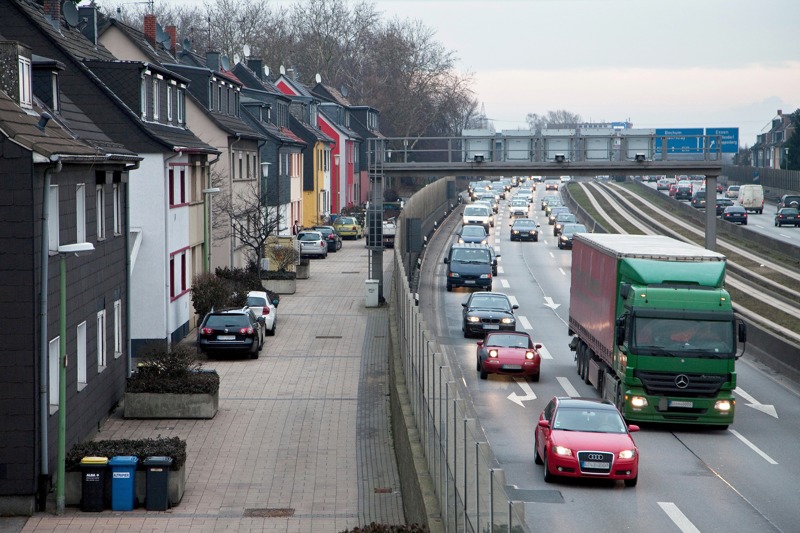
Homes bordering Ruhrschnellweg, a highway in Essen, Germany. © imageBROKER/Alamy

It’s not surprising that airborne particles dominated the disease burden, says C. Arden Pope III, an environmental economist at Brigham Young University who was not involved in the current study. “Everyone is exposed to particulates. These findings reflect the ubiquity of that exposure, even in developed countries, compared to other risk factors such as radon or even secondhand smoke, where exposures may be more limited,” he says.

The World Health Organization (WHO) has estimated that outdoor air pollution was responsible for 3.7 million deaths in 2012—roughly 1 in 15 deaths globally.^3^ Most of those deaths were in low- and middle-income countries that often struggle with high levels of ambient air pollution. However, the current analysis suggested PM_2.5_ was the dominant risk factor even in Finland, a clean and affluent country by WHO standards, and the country with the lowest air pollution impacts of the six studied. “We found PM to be roughly twice as harmful as fatal traffic accidents in Finland,” Hänninen says.

Also surprising, he says, was the evidence of a high impact of traffic noise, which the authors evaluated as a risk factor for severe sleep disturbance and ischemic heart events. The researchers estimated that traffic noise cost Europeans 400–1,500 years of life per million people due to death and disability. “We actually expect that estimate for noise to be too low,” Hänninen says, because the researchers did not include all known health end points for noise exposure, such as hypertension and heart disease.^4^

The researchers were able to obtain exposure data collected with standardized methods in all countries only for secondhand smoke, PM_2.5_, and ozone. They excluded suspected health effects that weren’t sufficiently researched or monitored. For instance, they did not account for developmental impacts of endocrine disruption, which have been associated with dioxin and PCB exposures.^5^

“The study is ambitious in its attempt to integrate the literature and apply it to an area of real concern,” Pope says. “The limitations of the paper reflect the limitations of our own knowledge.” The next important step, says Hänninen, is to identify preventable risks and then analyze potential policies for feasibility and cost effectiveness.
